# An experimental dataset on yields of pulses across Europe

**DOI:** 10.1038/s41597-023-02606-0

**Published:** 2023-10-17

**Authors:** Daniele Antichi, Silvia Pampana, Lorenzo Gabriele Tramacere, Véronique Biarnes, Ina Stute, Žydrė Kadžiulienė, Becky Howard, Isabel Duarte, Oskars Balodis, Iris Bertin, David Makowski, Nicolas Guilpart

**Affiliations:** 1 Department of Agriculture, Food and Environment, University of Pisa, Via del Borghetto 80, Pisa, 56124 Italy; 2 Centre for Agri-environmental Research “Enrico Avanzi”, University of Pisa, Via Vecchia di Marina 2, San Piero a Grado, 56122 Italy; 3 Terres Inovia, Avenue Lucien Bretignières, Campus de Grignon, Thiverval-Grignon, 78850 France; 4 Fachhochschule Südwestfalen, Lübecker Ring 2, Soest, 59494 Germany; 5 Lithuanian Research Centre for Agriculture and Forestry, Instituto al. 1, Akademija, Kėdainiai, LT-58344 Lithuania; 6 PGRO Research Limited, The Research Station, Great North Road, Thornhaugh, Peterborough, PE8 6HJ UK; 7 Instituto Nacional de Investigaçao Agraria e Veterinaria, Estrada de Gil Vaz, Apartado 6, 7351-901 Elvas, Portugal; 8 Faculty of Agriculture, Latvia University of Agriculture, Lielâ iela 2, Jelgava, LV-3001 Latvia; 9 Université Paris-Saclay, AgroParisTech, INRAE, UMR Agronomie, 91120 Palaiseau, France; 10 University Paris-Saclay, AgroParisTech, INRAE, UMR MIA Paris-Saclay, 91120 Palaiseau, France

**Keywords:** Agroecology, Ecosystem services, Plant ecology

## Abstract

Future European agriculture should achieve high productivity while limiting its impact on the environment. Legume-supported crop rotations could contribute to these goals, as they request less nitrogen (N) fertilizer inputs, show high resource use efficiency and support biodiversity. However, legumes grown for their grain (pulses) are not widely cultivated in Europe. To further expand their cultivation, it remains crucial to better understand how different cropping and environmental features affect pulses production in Europe. To address this gap, we collected the grain yields of the most cultivated legumes across European countries, from both published scientific papers and unpublished experiments of the European projects LegValue and Legato. Data were integrated into an open-source, easily updatable dataset, including 5229 yield observations for five major pulses: chickpea (*Cicer arietinum* L.), faba bean (*Vicia faba* L.), field pea (*Pisum sativum* L.), lentil (*Lens culinaris* Medik.), and soybean (*Glycine max (L.)* Merr.). These data were collected in 177 field experiments across 21 countries, from 37° N (southern Italy) to 63° N (Finland) of latitude, and from ca. 8° W (western Spain) to 47° E (Turkey), between 1980 and 2020. Our dataset can be used to quantify the effects of the soil, climate, and agronomic factors affecting pulses yields in Europe and could contribute to identifying the most suitable cropping areas in Europe to grow pulses.

## Background & Summary

Grain legumes (also named pulses) are Fabaceae crops sown and harvested for dry grain production and used as feed or food. Nowadays, they are widely recognized as key components of sustainable cropping systems, because, besides their use as food and feed, they may additionally deliver several supporting and regulating ecosystem services^1,2^.

Legumes have the unique ability to establish a symbiosis with rhizobia bacteria to fix atmospheric N_2_, thus providing another source of nitrogen (N) to the plant in addition to that available in soils. Moreover, the mineralization of their N-rich crop residues enlarges soil N availability for the following crops^3,4^. Together, these processes can reduce mineral N fertilizer requirements in cropping systems, and consequently can lower fossil energy use and direct/indirect net greenhouse gas (GHG) emissions in agriculture, associated with the manufacture and field application of mineral N fertilizers^4^. Moreover, increased cultivation of grain legumes would contribute to the diversification of cereal-dominated crop rotations in Europe, with expected positive effects on biodiversity, control of weeds, pests and diseases, and soil structure^5,6^, as well as on crop yields^7^.

Legumes can also have negative outcomes. For example, higher soil N losses to the environment have been observed due to the higher soil N content after legume harvest^8,9^. In comparison to cereals, some legume crops may also require a higher pesticide use intensity (e.g., pea) or more irrigation water (e.g., soybean)^10,11^. However, despite these potential drawbacks, the overall benefits of increased legume cultivation in the European Union (EU) are still expected to be positive^9,12^. Nevertheless, the presence of legumes in cropping systems of the EU is still scarce (nowadays, about 2% of arable land) so that the EU is highly dependent on imports of feed-legumes and agricultural systems are still heavily reliant on fossil energy used for the manufacture of synthetic nitrogen fertilizers^8,13^.

Several subsidies have been introduced under the EU’s Common Agricultural Policy (CAP) and Rural Development Plans (RDPs) to increase the presence of grain legumes in European farming systems, to support both the transition to more sustainable food production^9^ and the goals of the European Green Deal “Farm to Fork” strategy^14^. Nevertheless, many socio-economic and agronomic factors have been shown to explain the marginalization of grain legumes in Europe^15,16^. Importantly, annual economic margins of pulses remain too low in comparison to other crops like cereals, due to inferior yields, higher yield instability^17^, and lower market prices^18^. Moreover, investments in research and development (R&D) have been lower for legumes than for other major crops, and legume cultivation is facing many genetic and agronomic challenges that need to be addressed to improve the yield and yield stability of pulses. These challenges include breeding for improved varieties^19^, as well as abiotic stress management^20,21^ and the control of pests, diseases, and weeds, which is particularly challenging if synthetic agrochemicals cannot be used like in organic farming systems^22^.

To increase the growth of grain legumes in Europe, an essential first step is to identify the most suitable areas for their cultivation, i.e., regions where high and stable yields can be attained. Indeed, a better knowledge of the most suitable areas for pulses is important to identify which legume species are best adapted to local conditions of climate and soils^23^, identify the most important limiting factors and improve agronomic management of these crops across a wide range of agroclimatic zones in Europe^1,24^, and give guidance for the development of value chains. To date, European regions potentially relevant to grow pulses have been only identified for soybean^25^, but not for other grain legumes. Moreover, many different cultivars were produced for a given species and they could respond differently to different environments and management practices (Genotype x Environment x Management interactions^26^). It is thus important to build large datasets including yield data covering a wide range of cultivars, environments, and management practices to robustly identify suitable areas^23,25,27^.

Some existing datasets provide yield data for grain legumes in Europe, but none of them meets all the characteristics required for our purpose. For example, annual yield data of grain legumes are provided at the country level by the Food and Agriculture Organization (FAO) of the United Nations (see https://www.fao.org/faostat/en/#data), but the spatial resolution is too coarse to capture local effects of variations in climate and soils on crop yield. The Global Dataset of Historical Yields for major crops provides annual yield data at a spatial resolution of 0.5° (grid cells of 55 km), but only for soybean^28,29^. The Spatial Production Allocation Model (SPAM) data provide yield data for 42 crops including grain legumes at the global scale at a spatial resolution of 0.083°, with 10-km grid cells, but only for the year 2010^30^. Moreover, available datasets mostly provide yield data where crops are grown, while observed actual yields in areas where grain legumes are not yet grown by farmers would be of interest as well, to identify areas where grain legumes could be potentially grown in the future. A previous dataset of legume yields from field experiments was made available by Cernay and co-authors^27^. It covers the period 1967–2016 and includes records across 41 countries and 18 Köppen-Geiger climatic zones. But this dataset is focused on field experiments comparing several pulse species and does not include yield data collected in experiments including single pulse species. This dataset thus missed numerous experimental pulse yield data obtained in Europe.

To fill this gap, we present a new dataset on grain legume yields, gathering results from field experiments for the five dominant species in Europe: chickpea (*Cicer arietinum* L.), faba bean (*Vicia faba* L.), field pea (*Pisum sativum* L.), lentil (*Lens culinaris* Medik.), and soybean (*Glycine max (L.)* Merr.). This dataset, named the “European Grain Legume Dataset” (EGLD) includes 5229 yield observations from 177 field experiments across 21 countries, from 1980 to 2020. EGLD can prove to be useful for disentangling the effects of soil, climate and agronomic drivers of legume yields in Europe.

## Methods

**Fig. 1:**
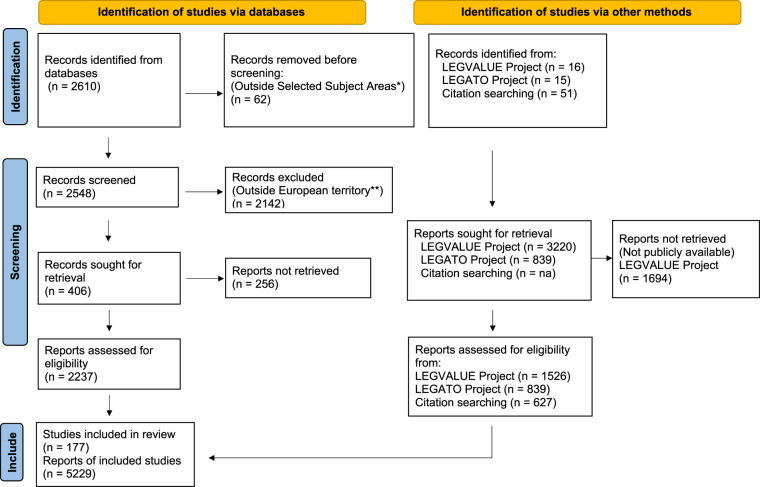
PRISMA^186^ flow diagram for the systematic review process, including searches of database and of unpublished experiments of LEGVALUE and LEGATO Projects. *EU-27 countries plus Turkey, Albania, Switzerland, United Kingdom, Norway, Ukraine, Serbia, Montenegro, North Macedonia, Kosovo, Moldova, Iceland, Belarus) ** “Agricultural and Biological Sciences” and “Environmental Sciences”.

Data were collected from three different sources (Fig. 1): (i) published experimental data from an online literature search; (ii) experimental data from trials conducted within the EU-FP7 LEGATO (LEGumes for the Agriculture of TOmorrow) Project (GA nr. 613551); (iii) experimental data from trials conducted within the EU-H2020 LEGVALUE (Fostering sustainable legume-based farming systems and agri-feed and food chains in the EU) Project (GA nr. 727672). Five pulses, namely chickpea, faba bean, field pea, lentil, and soybean have been searched, as the most cultivated in Europe.

### Data collection from scientific papers

Concerning the first source of information, we used the Scopus collection to retrieve the foremost publications reporting peer-reviewed research on the selected pulse species. The systematic search of peer-reviewed journals was completed in June 2020. The search criteria included the following elements, searched for in the following order within article title, abstract, and keywords:

- Crop*
- AND one of the following terms: chickpea, faba bean, lentil, pea, soybean
- AND (yield OR ‘dry matter’ OR biomass)
- AND (compar* OR assessment OR product* OR performance*)
- AND (trial* OR factorial OR experiment* OR treatment* OR condition*).

At first, we did not set any restrictions on publication date and language and retrieved 2610 papers. The search results were refined to “Agricultural and Biological Sciences” and “Environmental Sciences” as subject areas in Scopus. After that, we further restricted the records to affiliation countries in Europe (EU-27 countries plus Turkey, Albania, Switzerland, United Kingdom, Norway, Ukraine, Serbia, Montenegro, North Macedonia, Kosovo, Moldova, Iceland, and Belarus).

At that point, the literature search identified 406 articles of potential interest (Fig. 1).

We also gathered 51 additional papers from the literature review by Ditzler *et al*.^1^ and from the references cited in the eligible papers identified above, that had not been identified by the initial search, according to our inclusion criteria.

Article titles and abstracts have then been screened for eligibility according to the following criteria: (i) article title and/or article abstract reporting one or more grain legume species grown as sole crop; (ii) article title and/or article abstract reporting at least one experiment at one of the countries listed above; (iii) article title and article abstract published in a peer-reviewed journal. All the eligible full-text articles were thoroughly read at least twice and by two different authors to assess their admissibility according to additional criteria: (i) at least one grain legume grown as sole crop per each experimental site included in the field experiment; (ii) data coming from experimental trials; (iii) study reporting grain yields (i.e., papers reporting only total plant biomass were excluded); (iv) precise information on field site (region and site name and/or latitude and longitude coordinates); (v) main agronomic practices reported. At least the year was considered a mandatory information for sowing and harvest to relate the agronomic data with weather conditions. If the month of sowing/harvest, but not the day, was reported, then the central day of the month was used as a proxy of the actual sowing/harvest dates. Then, if grain yield data were expressed as fresh matter without indication of percent moisture, they were excluded; otherwise, fresh matter data were converted into dry matter and all values were reported as dry weights per unit area.

We finally picked out 146 eligible full-text articles published between 1990 and 2020^31–176^. Data were manually extracted from the tables of the selected papers and the WebPlotDigitizer-4.2.0 app (https://automeris.io/WebPlotDigitizer) was used to extract data from figures. All data were converted into a digital format and inserted into a csv file.

### Data collection from experimental trials

To expand the dataset, we sought out further yield data that met the following criteria: i) data coming from experimental trials; ii) at least one of the selected grain legume species grown as sole crop; iii) experimental site of the field experiment at one of the countries listed above; iv) study reporting grain yields; v) disposal of precise information on field site (region and site name and/or latitude and longitude coordinates); vi) main agronomic practices available and reported. Accordingly, we included also open data provided by the former EU-FP7 project LEGATO “LEGumes for the Agriculture of TOmorrow” (2014–2017), which has delivered the results of the 2-yr experimental activities performed on field pea, faba bean, chickpea, and lentil. The methodological aspects and the experimental details of the trials are openly available at https://intranet.iamz.ciheam.org/forms/Legato/WP6/files/Field_Trial_Protokol_5.1.2016.pdf. The dataset was downloaded from https://intranet.iamz.ciheam.org/forms/Legato/WP6/index.php and eligible data, according to the criteria, were extracted and manually added to the dataset. Overall, data were produced in field experiments in compliance with a common protocol. The field experiments were conducted on plots of 10 m^2^ and replicated four times in space, according to a completely randomized design (CRD). Plant samples for grain yield assessment were collected on 1 m^2^ sampling areas when the crops reached harvest maturity.

The dataset was also complemented with the European H2020 LEGVALUE Task 1.2 Partners (www.legvalue.eu) own data, which were generated both on-station and on-farm but always under well-determined field conditions. The authors, as effective partners of the Project could deliver their not already published results. Data that met the eligibility criteria were added by each partner to the original dataset, following the related instructions (see Data records section). The trials were conducted according to site-specific experimental protocols, that were documented by the providing partners and are available in the dataset (Supplementary Table S1).

Those details on geographical site, pedoclimatic conditions, treatment replications, and agronomic management (i.e., cultivar choice, tillage, plant density, fertilization, crop protection, growing cycle length) have been included in the dataset for each entry coming from both LEGATO and LEGVALUE experiments.

Additional information on the experimental methodology of the trials of both LEGATO and LEGVALUE are summarized in Supplementary Table S2.

## Data Records

The dataset created with all the extracted data, named “European Grain Legumes Dataset” (EGLD) is available at figshare^177^.

### Overview of data files

In the figshare repository, the following files are provided^177^: (i) “European Grain Legume Dataset.csv” that contains the data; (ii) “EGLD instructions.pdf” and “EGLD instructions.csv” that report the list and the definition of each column in the dataset (meta-data); (iii) “List of sources.pdf” and “List of sources.csv” that provide the list of the sources where the data were collected from.

In “EGLD instructions” files we reported a brief description and the assumptions for each column (category) for the reader’s guidance, to facilitate data entry operations as well as to inform the proper interpretation of the data.

The files “List of sources” complement information about the founts from which the data has been acquired, together with the web link at which they are accessible and the responsible institution.

### Overview of the structure of the dataset

The “European Grain Legume Dataset.csv” file contains all the data for the five selected pulse species, with variables as columns and entries as rows. As several papers and experiments reported multiple yield assessments, a unique ID was assigned to a single combination of year x site x crop species x experimental treatment level and was reported as a single entry (i.e., a single row) in the dataset.

**Fig. 2:**
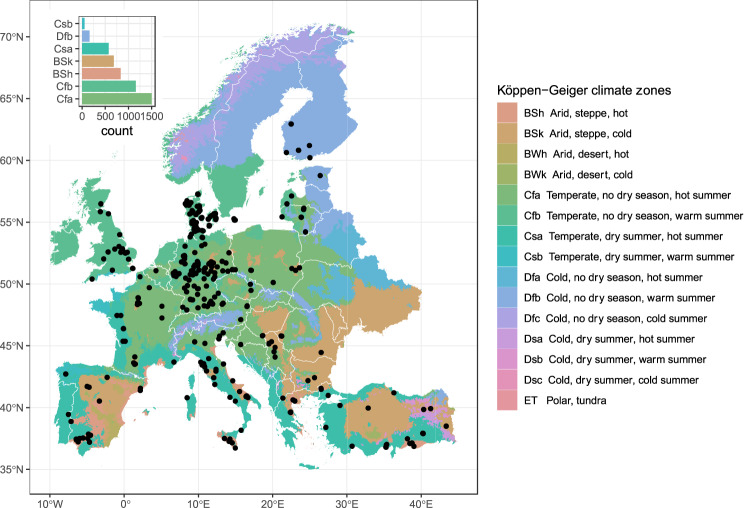
Geographic distribution of the experimental sites reporting the yields of the five pulse species. The insert provides the number of data in the seven most represented Koppen Geiger climate zones^186,187^.

This process led to a total of 5229 yield data for the five selected pulses all over Europe (Table 1 and Fig. 2), of which 2864 were collected from published papers, 1526 from non-published experimentations of the LEGVALUE project, and 839 from the LEGATO project. Records identified from published papers or the LEGATO Project or other experiments of the LEGVALUE Project were differently labeled in the database in the column “source” as Paper, Experiment LEGATO or Experiment LEGVALUE, respectively. The coordinates of each yield data were extracted and included in “European Grain Legume Dataset.csv”.

**Table 1:** Distribution of dataset entries among European Union countries for the five pulse species included in EGLD.

Country	Chickpea		Faba bean		Field pea		Lentil		Soybean		Total (country)
	n	*(%)*	n	*(%)*	n	*(%)*	n	*(%)*	n	*(%)*	n
Austria	2	*(0.4)*	48	*(2.8)*	52	*(3.7)*			6	*(0.5)*	108
Belgium									14	*(1.2)*	14
Bulgaria	4	*(0.9)*			24	*(1.7)*					28
Croatia									24	*(2.0)*	24
Czech Republic			38	*(2.2)*	66	*(4.7)*					104
Denmark	4	*(0.9)*	75	*(4.3)*	113	*(8.0)*	4	*(1.0)*	4	*(0.3)*	200
Estonia			38	*(2.2)*	38	*(2.7)*					76
Finland			38	*(2.2)*	51	*(3.6)*					89
France			212	*(12.2)*	150	*(10.6)*			44	*(3.7)*	406
Germany			661	*(38.0)*	331	*(23.4)*	58	*(14.0)*	486	*(40.5)*	1536
Greece	168	*(36.3)*	20	*(1.1)*	36	*(2.5)*	137	*(33.2)*			361
Italy	85	*(18.5)*	186	*(10.7)*	63	*(4.4)*	25	*(6.1)*	88	*(7.3)*	447
Latvia			5	*(0.3)*	1	*(0.1)*			5	*(0.4)*	11
Lithuania			5	*(0.3)*	31	*(2.2)*			24	*(2.0)*	60
Poland			7	*(0.4)*	39	*(2.8)*			57	*(4.8)*	103
Portugal	51	*(11.1)*	20	*(1.1)*	26	*(1.8)*					97
Romania					12	*(0.8)*	24	*(5.8)*	82	*(6.8)*	118
Serbia			12	*(0.7)*	34	*(2.4)*			147	*(12.3)*	193
Spain	112	*(24.4)*	161	*(9.2)*	90	*(6.4)*	49	*(11.9)*			412
Turkey	33	*(7.2)*			70	*(4.9)*	116	*(28.1)*	218	*(18.2)*	437
United Kingdom			215	*(12.3)*	190	*(13.4)*					405
**Total (crop)**	**459**	—	**1741**	—	**1417**	—	**413**	—	**1199**	—	**5229**

n = number of entries and (%) = percent of entries by country.

All the sources from which data were recovered are reported in the file named “List of Sources.pdf”, available on the figshare repository^177^. For data obtained from the LEGATO project or from experiments of the LEGVALUE project, a brief description of the experiment is delineated, while for published papers the full reference is reported. We also provide a web address where the sources can be accessed (through the digital identification number (doi) if available). Most of the published papers are publicly available under Open Access licenses. A few can be accessed by personal or institutional subscription, depending on each journal policy.

All records enclose 72 columns that describe a corresponding number of variables with different types of information. These variables can be grouped into four categories: the first provides information on the source of the data, the second on the experiment, the third on agricultural management practices, and the fourth category conveys figures about crop yield. The full list of variables included in the dataset, together with their detailed description and unit has been reported in Supplementary Table 1.

### Overview of the data

The EGLD^177^ contains yield data from 21 countries, from ca. 37° N (southern Italy) to 63° N (Finland) of latitude, and from ca. 8° W (western Spain) to 47° E (Turkey) (Fig. 2), thus capturing a wide range of pedoclimatic conditions. With about one third (29%) of the data, Germany is the country with the highest number of observations, followed by Italy, Turkey, Spain, France, the UK, and Greece, each of these countries representing about 8% of the dataset (Table 1). Other countries have lower numbers of observations, with Bulgaria, Croatia, Belgium, and Latvia having less than 30 observations each.

**Fig. 3:**
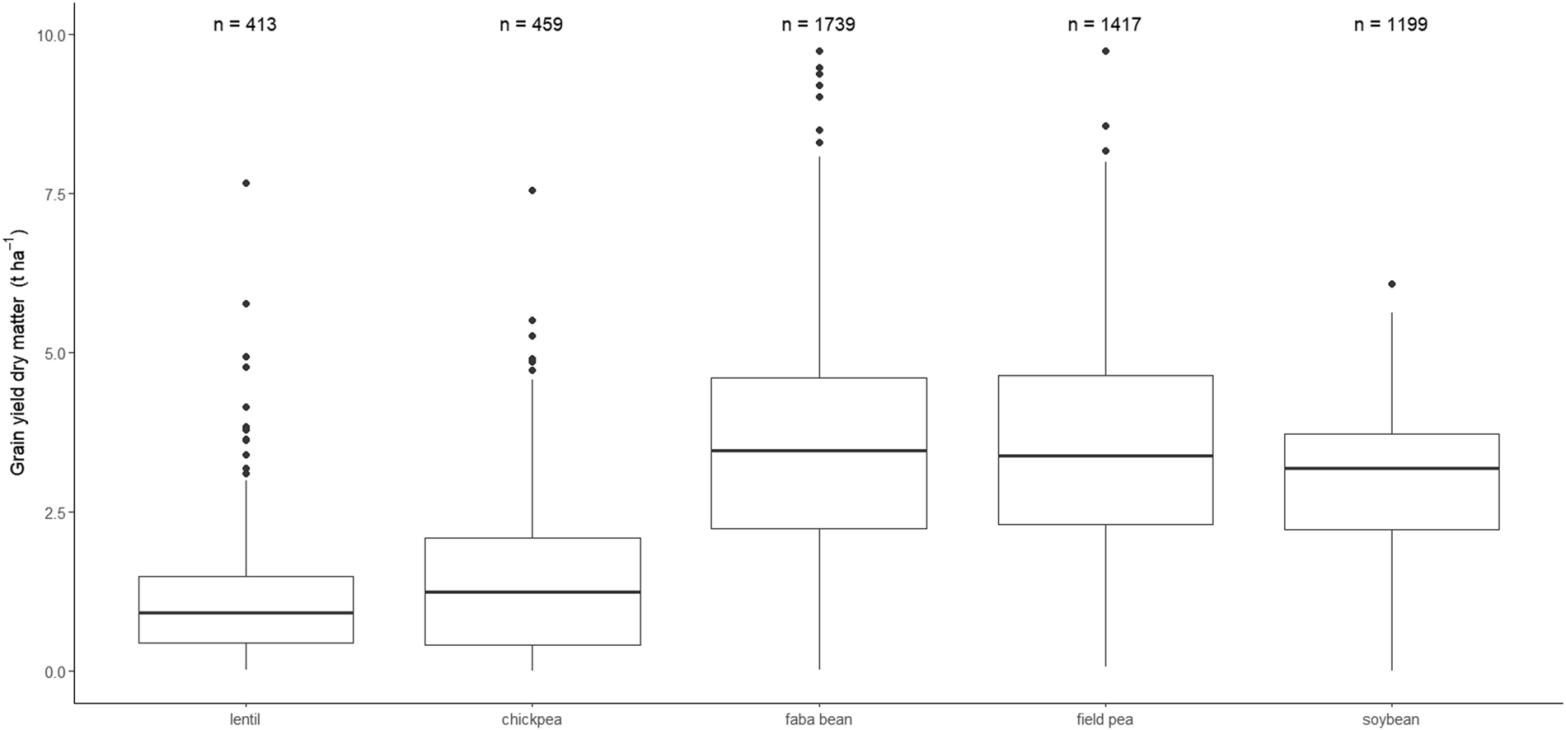
Box plots showing the distributions of grain yield data for the five pulse species. Values are reported as dry weights per unit area (t ha^−1^). Main body of the boxplot shows the interquartile range (IQR = Q3-Q1), and the central line the median (Q2). Whiskers (bars) represent Q1-1.15 IQR (lower) and Q3 + 1.5 IQR (upper), and dots indicate outliers.

Faba bean, pea, and soybean are well represented in the dataset as they account for 33%, 27%, and 23% of total number of observations, and are present in 19, 16, and 13 of the 21 countries, respectively. On the other hand, chickpea and lentil are much less represented as they account for only 9% and 8% of the whole dataset, and are present in 8 and 7 countries, respectively. Belgium and Croatia provide information only for one pulse (soybean), while Italy and Denmark have information about all the five species. The dataset captures a wide range of grain yield values, from complete crop failure (yield almost null) to very high yield (for all the species, the maximum yield was higher than 6 t ha^−1^ of dry matter) (Fig. 3). The most represented agronomic practice that has been evaluated in the experimental trials was found to be the genotype (cultivar) as it represents more than 50% of all the entries of the dataset. Tillage and weed control are the next, with 8 and 6% of total entries, respectively.

Missing data are indicated as “NA” (not available) cells in the dataset. For example, cultivar was missing in 7% of entries, soil texture in 33% of the total, while the soil classification was not reported for more than half entries (about 62%), and tillage was missing in half (52%) of entries (Table 2).

**Table 2:** Number of entries with missing data about soil classification, texture, cultivar, plant and sowing density, and row spacing (per source of information and in total).

Source	Soil classification	Soil texture	Cultivar	Tillage	Plant density	Sowing density	Row spacing
LEGATO Experiment	801	357	0	581	139	173	839
LEGVALUE Experiment	878	202	100	1162	1316	460	1038
Paper	1563	1161	264	961	2432	974	839
*Total*	*3242*	*1720*	*364*	*2704*	*3887*	*1607*	*2716*

## Technical Validation

After the data extraction, the quality check was further carried out by comparing the entire set of collected data against the corresponding original source (papers or experiments) to make sure the data were digitalized correctly. The formats of each column (numerical or string) were checked to correct misprinting. We visualized the data distribution for each numerical column and detected outliers, that were manually checked and validated by comparing them with the values reported in the original papers or experiments. Moreover, the values of crop yield reported in the database are consistent with the results of published research^1,17,23,178–180^. Graphical exploration of the data was performed by using the package ggplot2 with the ggplot function of the statistical software R, version 4.1.2.

## Usage Notes

**Fig. 4:**
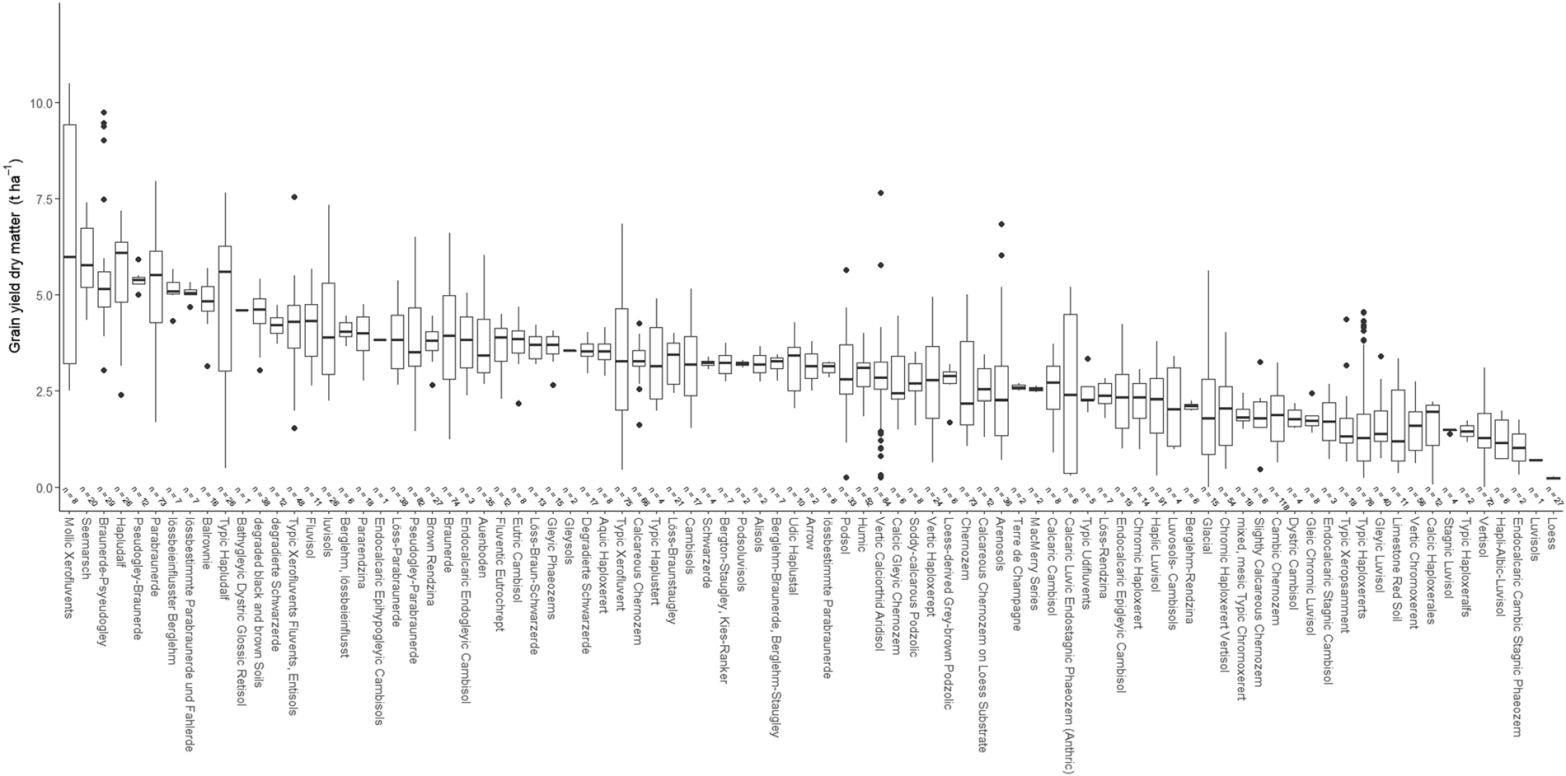
Box plots of grain yields dry matter (t ha^−1^), ranked by soil type. Main body of the boxplot shows the interquartile range (IQR), and the central line the median. Whiskers (bars) represent Q1-1.15 IQR (lower) and Q3 + 1.5 IQR (upper), with dots to indicate outliers. n = Number of entries. Values are pooled across the five species.

**Fig. 5:**
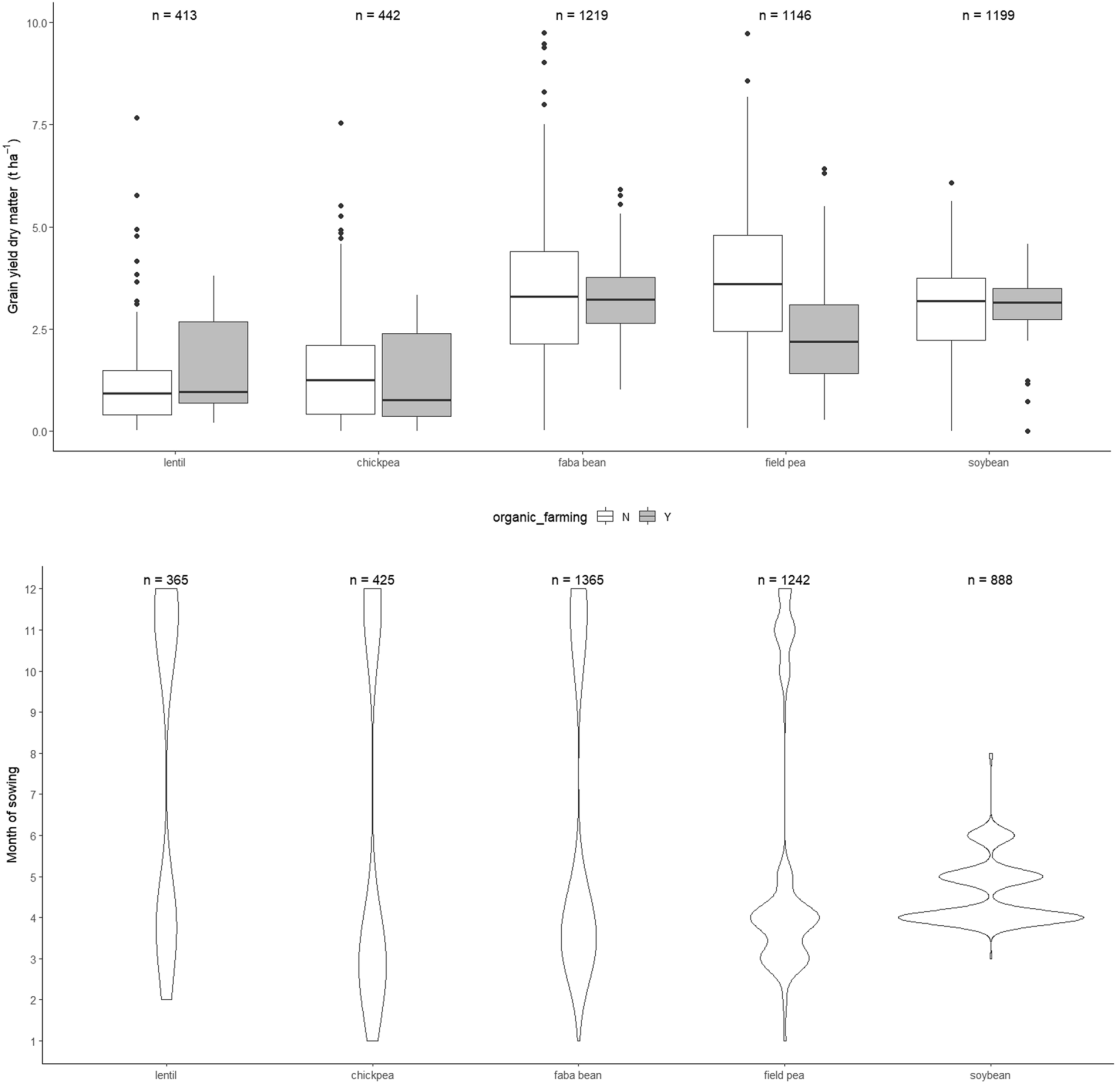
Two examples of management practices included in the dataset: organic cultivation and month of sowing. (**a**) Box plot of the effect of organic cultivation (grey) vs conventional cultivation (white) on the yield of the five pulse species. Main body of the boxplots shows the interquartile range (IQR), and the central line the median. Whiskers (bars) represent Q1-1.15 IQR (lower) and Q3 + 1.5 IQR (upper), with dots to indicate outliers. (**b**) Violin plot of the month of sowing for the five pulse species. The width of the areas represents the proportion of data located. n = Number of entries.

The yield dataset can be used for several purposes. First it is useful to improve our knowledge of the relationships between pedoclimatic factors, agronomic practices, and legume yield. The wide range of soil types (Fig. 4), climate zones (Fig. 1), and management practices (Fig. 5) covered by our dataset provides a unique opportunity to identify the main factors influencing yield of pulses^8,181,182^. A better understanding of GxExM interactions impacts of the yield for grain legumes would contribute to improving our understanding of the determinants of productive performances of the cultivars tested in Europe and identify the most promising in different regions^15,16^.

Second, the dataset can be used to map achievable yields of grain legumes over Europe under current and future climate scenarios. This can be done by fitting statistical or machine learning models to predict grain yield from climate (and possibly other factors like soil and management practices) inputs, as done by Guilpart *et al*. for soybean^25^. Thanks to the experiment geographical coordinates, it is possible to associate climate and soil information with the yield data included in our dataset^183–185^. It would be then possible to train data-driven yield forecasting models and produce yield maps for the five pulse species under various hypothetical climate and management scenarios, as done for soybean^25^. Such yield maps may be highly relevant to: (i) identify suitable areas for pulses cultivation in Europe under both current and future climate; (ii) simulate the impact of an increase of pulses growing area on the pulse production in Europe; (iii) support the definition of market scenarios as well as policies on protein/starch production in the EU, highlighting potentialities, environmental barriers, and constraints; (iv) identify pulses species and cultivars best adapted to local pedoclimatic conditions over Europe.

Notably, the EGLD dataset could be easily updated using data retrieved from recently published papers and data recorded in new experiments. However, at the figshare accession data is that peer reviewed in 2023^177^ and this version will be maintained.

### Supplementary information

Supplementary Table S1

Supplementary Table S2

## Data Availability

Prisma Flow Diagram (http://www.prisma-statement.org/documents/PRISMA_2020_flow_diagram_new_SRs_v2.docx) was used to generate Fig. 1. The statistical software R (version 4.1.2, 2021-11-01-“Bird Hippie” Copyright © 2021. The R Foundation for Statistical Computing) was used to produce Figs. 2–5. R scripts used to produce figures are openly available on the figshare repository.
