# Supplementary material for: An experimental dataset on yields of pulses across Europe
Source: Sci Data. 2023 Oct 17;10:708. doi: 10.1038/s41597-023-02606-0 (PMC10582191; doi:10.1038/s41597-023-02606-0)
Supplement: Supplementary file 1 — Supplementary Table S1 [file 41597_2023_2606_MOESM1_ESM.docx]

**Supplementary Table S1. Metadata of the European Legume dataset.**

| **Category** | **Column** | **Name** | **Data collected** | **Unit** | **Notes** |
| --- | --- | --- | --- | --- | --- |
| **General information on the Source** | A | **id** | ID of the Entry |  | Progressive number |
|  | B | **source** | Type of source |  | Paper or Experiment LEGATO or Experiment LEGVALUE |
|  | C | **experiment_ID** | ID of the Experiment/Paper |  | Experiment acronym_Surname of responsible or FirstAuthorSurnameYEAR |
| **Information on the Experiment** | D | **site_country** | Name of the Country in full |  |  |
|  | E | **site_region** | Name of the Region in full |  | NA if not available |
|  | F | **site_name** | Name of the Site in full |  | NA if not available |
|  | G | **lat** | Decimal degrees of Latitude with two digits (xx.xx) | degrees | NA if not available |
|  | H | **lon** | Decimal degrees of Longitude with two digits (xx.xx) | degrees | NA if not available |
|  | I | **site_soil_classification_name** | Soil classification type (according to USDA) |  | NA if not available |
|  | J | **site_soil_texture_name** | Soil texture class |  | e.g. loam, sandy, sandy loam NA if not available |
|  | K | **soil_texture_anomaly** | Soil texture reported is not standard | Y/N |  |
|  | L | **site_rain** | Total rainfall in the period considered | mm | NA if not available |
|  | M | **site_rain_period** | annual/growing season |  | NA if not available |
|  | N | **site_rain_period_month** | Initial Final month of the period for which rainfalls are reported |  | e.g. Jan Dec - NA if not available |
|  | O | **site_rain_period_year** | Year of rainfall registration |  | e.g. 1993 NA if not available |
|  | P | **site_temp** | Average temperature in the period | °C | NA if not available |
|  | Q | **site_temp_period** | Annual/Growing Season |  | NA if not available |
|  | R | **site_temp_period_month** | Initial Final month of the period for which temperature is reported |  | e.g. Jan Dec - NA if not available |
|  | S | **site_temp_period_year** | Years of the temperature registration |  | e.g. 1993 NA if not available |

| **Category** | **Column** | **Name** | **Data collected** | **Unit** | **Notes** |
| --- | --- | --- | --- | --- | --- |
| **Information about agricultural management activities** | T | **organic_farming** | Organic techniques applied or not | Y/N |  |
|  | U | **management_evaluated** | Agronomic management evaluated in the experiment |  | e.g. tillage, irrigation, variety |
|  | W | **treatment_name** | Report the name of the treatment or (in case of factorial combination) the name of the combination |  |  |
|  | X | **scientific_name** | Latin name (without author initials) of the legume crop species |  | e.g. Glycine max |
|  | Y | **previous_crop** | Latin name (without author initials) of the crop species grown before the legume |  | e.g. Triticum aestivum NA if not available |
|  | Z | **crop** | Common name of the legume crop species |  | e.g. Soybean |
|  | AA | **crop_type** | Type of the crop |  | Pea: "green" or "dry" Faba bean: "horse" for var. equina, "pigeon" for var. minor, "broad" for var. major |
|  | AB | **cultivar** | Name of the legume crop variety |  |  |
|  | AC | **precocity_group** | Precocity of the group (only for soybean) | 000 to 10 | NA if not available |
|  | AD | **gm** | Genetically modified variety | Y/N | Yes if genetically modified, No if not |
|  | AE | **sow_dd** | Day of sowing as originally reported in the source document. If more than one, the range is reported |  | NA if not available |
|  | AF | **sow_mm** | Month of sowing as originally reported in the source. If more than one, the range is reported |  | NA if not available |
|  | AG | **sow_yy** | Year of sowing as originally reported in the source. If more than one, the range is reported |  | NA if not available |
|  | AH | **sow_date** | Date of sowing | mm/dd/yyyy | NA if not available |
|  | AI | **har_dd** | Day of harvest as originally reported in the source document. If more than one, the range is reported |  | NA if not available |
|  | AJ | **har_mm** | Month of harvest as originally reported in the source document. If more than one, the range is reported |  | NA if not available |
|  | AK | **har_yy** | Year of harvest as originally reported in the source document. If more than one, the range is reporte |  | NA if not available |
|  | AL | **har_date** | Date of harvest | mm/dd/yyyy | NA if not available |
|  | AM | **cycle_length** | Length of crop cycle in the experimental year | days | nr. of days from sowing to harvest  NA if not available |
|  | AN | **tillage** | Tillage performed or not | Y/N | Yes if a tillage operation is performed before legume sowing, or Not if sod-seeding legume NA if not available |
|  | AO | **plant_density** | Number of legume plants per unit area (alternative to sowing density) | n m-2 | NA if not available |
|  | AP | **sowing_density** | Number of legume seeds per unit area (alternative to plant density) | n m-2 | NA if not available |
|  | AQ | **row_spacing** | Inter-row distance | m | NA if not available |
|  | AR | **N_rate** | Total amount of N applied to the crop | kg N ha-1 | NA if not available |
|  | AS | **N_fertiliser type_1** | Name(s) of the first N fertiliser applied to the crop with N application rate | kg N ha-1 | e.g. Poultry manure -30-  NA if not available NR if not relevant (if not applied) |
|  | AT | **N_fertiliser type_2** | Name(s) of the second N fertiliser applied to the crop with N application rate | kg N ha-1 | e.g. Poultry manure -30-  NA if not available NR if not relevant (if not applied) |
|  | AU | **N_fertiliser type_3** | Name(s) of the third N fertiliser applied to the crop with N application rate | kg N ha-1 | e.g. Poultry manure -30-  NA if not available NR if not relevant (if not applied) |
|  | AV | **N_nb_application** | Number of applications of N fertilisers | n | NA if not available NR if not relevant (if not applied) |
|  | AW | **N_perc_from_organic_fert** | Percent of total N supplied to the crop coming from organic fertilisers or amendments | % | NA if not available NR if not relevant (if not applied) |
|  | AX | **P_rate** | Total amount of P supplied to the crop | kg P ha-1 | NA if not available |
|  | AY | **P_fertiliser_type_1** | Name(s) of the first P fertiliser applied to the crop with P application rate | kg P ha-1 | NA if not available |
|  | AZ | **P_fertiliser_type_2** | Name(s) of the second P fertiliser applied to the crop with P application rate | kg P ha-1 | NA if not available |
|  | BA | **P_nb_application** | Number of applications of P fertilisers | n | NA if not available NR if not relevant (if not applied) |
|  | BB | **P_perc_from_organic_fert** | Percent of total P supplied to the crop coming from organic fertilisers or amendments | % | NA if not available NR if not relevant (if not applied) |
|  | BC | **K_rate** | Total amount of K supplied to the crop | kg K ha-1 | NA if not available |
|  | BD | **K_fertiliser_type_1** | Name(s) of the first K fertiliser applied to the crop with its level of K application rate | kg K ha-1 | e.g. Poultry manure -30- NA if not available NR if not relevant (if not applied) |
|  | BE | **K_fertiliser_type_2** | Name(s) of the second K fertiliser applied to the crop with its level of K application rate | kg K ha-1 | e.g. Poultry manure -30- NA if not available NR if not relevant (if not applied) |
|  | BF | **K_nb_application** | Number of applications of K fertilisers | n | NA if not available NR if not relevant (if not applied) |
|  | BG | **K_perc_from_organic_fert** | Percent of total K supplied to the crop coming from organic fertilisers or amendments | % | NA if not available NR if not relevant (if not applied) |
|  | BH | **irrigation** | Irrigation applied or not | Y/N | Y if irrigation was applied or N if not  Y_partial/full (partial if irrigation did not cover the full water need of the crop or full if it did it)  NA if not available |
|  | BI | **irrigation_quantity** | Mean amount of irrigation water applied | mm | exact amount or MIN-MAX value if a range is reported NA if not available NR if not relevant (if not applied) |
|  | BJ | **herbicide_application** | Chemical herbicides applied or not | Y/N | NA if not available |
|  | BK | **mechanical_weed_control** | Mechanical weeding applied or not | Y/N | NA if not available |
|  | BL | **crop_protection** | Crop protection products applied (including natural or biocontrol agents) | Y/N | NA if not available |
| **Information on Yield data** | BM | **replicate_nb** | Number of replicates concurring to the mean yield value reported in a single site x year combination | n | e.g. number of blocks or spatial replicates  NA if not available |
|  | BN | **site_nb** | Number of different sites considered as spatial replicates for computing the mean yield value reported, if mean yield values for each site are not available | n | NA if not available |
|  | BO | **year_nb** | Number of years concurring to the mean yield value reported, if single year mean yield values are not available | n | NA if not available |
|  | BP | **moisture_at_harvest** | Moisture percentage of the marketable yield as reported in the source | % | e.g. "13" for 13%  NA if not available |
|  | BQ | **yield** | Yield of the grain of the legume crop as dry matter | t d.m. ha-1 | The humidity reported in the previous colum is removed from the grain yield reported |
|  | BR | **yield_se** | Value of the standard error of the mean of the yield |  | NA if not available |
|  | BS | **yield_sd** | Value of the standard deviation of the mean of the yield |  | NA if not available |
|  | BT | **yield_cv** | Value of the coefficient of variation of the mean of the yield |  | NA if not available |
|  | BU | **yield_var** | Value of the variance of the mean of the yield |  | NA if not available |
