# Supplementary material for: An experimental dataset on yields of pulses across Europe
Source: Sci Data. 2023 Oct 17;10:708. doi: 10.1038/s41597-023-02606-0 (PMC10582191; doi:10.1038/s41597-023-02606-0)
Supplement: Supplementary file 2 — Supplementary Table S2 [file 41597_2023_2606_MOESM2_ESM.docx]

**Supplementary Table S2. Methodology adopted for grain legumes yield evaluation in the experimental trials of LEGATO and LEGVALUE Projects.**

| Project | Experiment ID | Country | Institution | Site | Experiment Type | Experimental Design | Plot size (m^2^) | Plant samples  (n plot^-1^) | Sampling area (m^2^) | Documents reporting methodological aspects * |
| --- | --- | --- | --- | --- | --- | --- | --- | --- | --- | --- |
| LEGATO | various | ES, IT, PT, EL, FR, DE, CZ, EE, SRB, UK, AT | Multiple (15) | Multiples (15) | On farm/on station | CRD | 10 | 4 | 1 | <https://intranet.iamz.ciheam.org/forms/Legato/WP6/files/Field_Trial_Protokol_5.1.2016.pdf>. |
| LEGVALUE | UNIPI | IT | UNIPI | Pisa | On-farm | CRD | 3,500-10,000 | 4 | 1 | NA |
|  | Ingvorsen | DK | SEGES | various | On-farm | CRD | 21 | 4 | 1 | NA |
|  |  | FR | TERIN | Poitou Charente | On-station | RSD | 1000 |  | 1 | NA |
|  | FH-SWF-FB | DE | FH-SWF | Soest | Literature | CRD | 10-20 | 4 | 1 | NA |
|  | Soja_LTU | LT | LRCAF | Akademija | On-station farm | RBD | 2000 | 4 | 1 (biometric analyses) 30 (yield) | <https://doi.org/10.3390/agronomy11020214> |
|  | LV | LV | AREI | Stende | Experimental farm | CRD | 21 | 1 | 10 | NA |
|  | CYTK- | PT | INIAV | Elvas | Experimental farm | CRD | 8 | 120 | 8 | NA |
|  | CPea | UK | PGRO | Nottinghamshire/ Lincolnshire | Experimental (on-farm) | RBD | 20 | - | 20 | NA |
|  | Fbean | UK | PGRO | Nottinghamshire/ Lincolnshire | Experimental (on-farm) | RBD | 20 | - | 20 | NA |
|  | Vpea | UK | PGRO | Nottinghamshire/ Lincolnshire | Experimental - farm scale | RBD | 20  10-20,000 | - | 20 | NA |

ES: Spain; IT: Italy; PT: Portugal; EL: Greece; EE: Estonia; FR: France; DE: Germany; CZ: Czech Republic; SRB: Serbia; UK: United Kingdom; AT: Austria; DK: Denmark; LT: Lithuania; LV: Latvia.

UNIPI: University of Pisa

SEGES: Danish Agricultural Research company

TERIN: Terres Inovia

FH-SWF: South Westphalia University of Applied Sciences

LRCAF: Lithuanian Research Centre for Agriculture and Forestry

AREI: Institute of Agricultural Resources and Economics

INIAV: Instituto Nacional de Investigação Agrária e Veterinária

PGRO: Processors and Growers Research Organisation

CRD: Completely Randomized Design; RBD: Randomized Block Design; RSD Repeated Strips Design

NA: not available

* Accessed on September 20, 2023
